# Supplementary material for: Implications for tetraspanin-enriched microdomain assembly based on structures of CD9 with EWI-F
Source: Life Sci Alliance. 2020 Sep 21;3(11):e202000883. doi: 10.26508/lsa.202000883 (PMC7536822; doi:10.26508/lsa.202000883)
Supplement: Supplementary file 1 [file LSA-2020-00883_Supplemental_Data_1.docx]

Supporting note 1

Diffuse scattering from CD9_EC2_ crystals.

Reciprocal space reconstructions were obtained from the diffraction images, using *IMG2HKL*, which is part of the EVAL package (Schreurs et al., 2010). Figure S3A shows three sections through reciprocal space. The crystal is twinned by a two-fold rotation along ***a*+b****. Figure S3B shows two twin domains with the twinning interface in the middle: a layer with base vectors c and a-b. Each layer is a possible twinning interface and diffuse streaks in the ***a*+b**** direction imply that the layers can also stack randomly. Starting from the middle layer, every fourth layer, belonging to the two twin structures, exactly overlap. Therefore, the structure can be indexed on a so-called stacking lattice (Dornberger-Schiff, 1956; Lutz and Kroon-Batenburg, 2018) with dimension 1/4c. On this lattice the twinned structure is completely ordered, causing reciprocal space reconstruction slices at l=4n to be ordered and all slices in between to have streaks in the direction ***a*+b****, i.e. the direction of packing disorder (Fig. S3B).
