# Supplementary material for: Implications for tetraspanin-enriched microdomain assembly based on structures of CD9 with EWI-F
Source: Life Sci Alliance. 2020 Sep 21;3(11):e202000883. doi: 10.26508/lsa.202000883 (PMC7536822; doi:10.26508/lsa.202000883)
Supplement: Reviewer comments [file LSA-2020-00883_review_history.pdf]

Life Science Alliance

Implications for tetraspanin-enriched microdomain assembly based on structures of CD9 with EWI-F

Wout Oosterheert, Katerina Xenaki, Viviana Neviani, Wouter Pos, Sofia Doulkeridou, Jip Manshande, Nicholas Pearce, Loes Kroon-Batenburg, Martin Lutz, Paul Van Bergen en Henegouwen, and Piet Gros
DOI: https://doi.org/10.26508/lsa.202000883

Corresponding author(s): Piet Gros, Utrecht University

Review Timeline:

Submission Date:	2020-08-18
Editorial Decision:	2020-08-21
Revision Received:	2020-08-25
Editorial Decision:	2020-09-03
Revision Received:	2020-09-04
Accepted:	2020-09-04

Scientific Editor: Shachi Bhatt

Transaction Report:

(Note: With the exception of the correction of typographical or spelling errors that could be a source of ambiguity, letters and reports are not edited. The original formatting of letters and referee reports may not be reflected in this compilation.)

Please note that the manuscript was reviewed at *Review Commons* and these reports were taken into account in the decision-making process at *Life Science Alliance.*

August 21, 2020

Re: Life Science Alliance manuscript #LSA-2020-00883-T

Prof. Piet Gros
Utrecht University
Dept. of Chemistry
Crystal and Structural Chemistry
Utrecht 3584 CH
Netherlands

Dear Dr. Gros,

Thank you for submitting your manuscript entitled "Implications for tetraspanin-enriched microdomain assembly based on structures of CD9 with EWI-F" to Life Science Alliance (LSA). We are pleased to say that we would like to invite you to submit a revised version that fits with LSA's formatting guidelines (http://www.life-science-alliance.org/authors).

For a brief overview: The manuscript was originally reviewed at Review Commons, and transferred to LSA with the reviews attached. The reviews were quite enthusiastic, and all three reviewers only pointed out minor edits which the authors had already addressed prior to submitting the manuscript to LSA.

Please cite https://doi.org/10.15252/embj.2020105246 in the manuscript, which was published while the manuscript was under review.

To upload the revised version of your manuscript, please log in to your account:
https://lsa.msubmit.net/cgi-bin/main.plex
You will be guided to complete the submission of your revised manuscript and to fill in all necessary information. Please get in touch in case you do not know or remember your login name.

We would be happy to discuss the individual revision points further with you should this be helpful.

While you are revising your manuscript, please also attend to the below editorial points to help expedite the publication of your manuscript. Please direct any editorial questions to the journal office.

The typical timeframe for revisions is three months. Please note that papers are generally considered through only one revision cycle, so strong support from the referees on the revised version is needed for acceptance.

When submitting the revision, please include a letter addressing the reviewers' comments point by point.

We hope that the comments below will prove constructive as your work progresses.

Thank you for this interesting contribution to Life Science Alliance. We are looking forward to

receiving your revised manuscript.

Sincerely,

Shachi Bhatt
Executive Editor
Life Science Alliance

A. THESE ITEMS ARE REQUIRED FOR REVISIONS

-- A letter addressing the reviewers' comments point by point.

-- An editable version of the final text (.DOC or .DOCX) is needed for copyediting (no PDFs).

-- High-resolution figure, supplementary figure and video files uploaded as individual files: See our detailed guidelines for preparing your production-ready images, http://www.life-science-alliance.org/authors

-- Summary blurb (enter in submission system): A short text summarizing in a single sentence the study (max. 200 characters including spaces). This text is used in conjunction with the titles of papers, hence should be informative and complementary to the title and running title. It should describe the context and significance of the findings for a general readership; it should be written in the present tense and refer to the work in the third person. Author names should not be mentioned.

B. MANUSCRIPT ORGANIZATION AND FORMATTING:

Full guidelines are available on our Instructions for Authors page, http://www.life-science-alliance.org/authors

We encourage our authors to provide original source data, particularly uncropped/-processed electrophoretic blots and spreadsheets for the main figures of the manuscript. If you would like to add source data, we would welcome one PDF/Excel-file per figure for this information. These files will be linked online as supplementary "Source Data" files.

IMPORTANT: It is Life Science Alliance policy that if requested, original data images must be made available. Failure to provide original images upon request will result in unavoidable delays in publication. Please ensure that you have access to all original microscopy and blot data images before submitting your revision.

We thank the three reviewers for providing insightful critiques on our manuscript.

Changes to document and comments made are marked e.g. "Reply 1.1" (referring the Reviewer #1 item #1, etc.) as described below.

Editorial comment

[E.1] Please cite https://doi.org/10.15252/embj.2020105246 in the manuscript, which was published while the manuscript was under review.

Reply E.1: We now cite the reference in both the introduction and discussion of the updated manuscript. The introduction states: "This was recently experimentally confirmed by the crystal structure of CD53, which showed an open conformation of the EC2 (Yang et al, 2020)", and the discussion states: "... more recently, full-length CD53 (Yang et al, 2020) , full-length CD9 ...".

Reviewer #1

I found this study to be very convincing. Prior studies are referenced appropriately, the text is well written and clear, the figures are clear also. In my opinion the paper does not need further experiment.

[1.1] The conclusions are well supported by the data. However, the concatenation model seems very speculative at this point. Also, it does not take into account the dynamics of these molecules.

Reply 1.1: The concatenation model combines the structural data from our manuscript with prior biochemical insights into tetraspanin homodimerization and with scanning-EM data on immunogold-labeled CD81 and CD9 on cells. It is not completely clear to us what reviewer #1 refers to with "the dynamics of these molecules". The cryo-EM data revealed that CD9 - EWI-F is a dynamic complex with straight and bent conformations, which could account for both circular and linear arrangements of tetraspanin-microdomains in cell membranes through the higher-order oligomerization of stable CD9 - EWI-F tetramers. Moreover, transient CD9 - CD9 interactions likely yield a variable number of complexes present in these concatenated and flexible strings of complexes. Such a concatenation model indeed requires further validation. However, it is consistent with experimental data and, importantly, provides a long-awaited molecular basis for TEM assembly. Although it was not within the scope of the current study, it will be of great interest to further investigate the concatenation model through detailed cell-biology based approaches.

Minor comment:

[1.2] There seems to be a mix up between the two structures in the following sentence p4: "In CD9EC2 - 4C8, the D loop adopts a partially helical conformation and central residue F176 is sandwiched by 4E8 residues W59 of CDR2 and W102 and R105 of CDR3 (Fig. 1D). In the 4C8-bound CD9EC2 structure the tip of the D loop points more outward and the Cα atom of F176"

Reply 1.2: The first sentence indeed mixed up the two structures and wrongfully mentioned $CD9_{EC2}$ - 4C8 instead of $CD9_{EC2}$ - 4E8. This has now been updated: "In $CD9_{EC2}$ - 4E8, the D loop adopts ..."

Reviewer #2

The paper is well written and the conclusions made are supported by the data presented.

[2.1] The ternary structure is in agreement with that of CD9 in complex with the related EWI-2 published earlier this year by Umeda et al (ref #25). The present work thus adds little structural

insights but may be useful in showing that the interaction pattern seen extends to another EWI protein family member.

Reply 2.1: We agree with reviewer #2 that that the CD9 - EWI-F structure presented in our work is similar to the CD9 - EWI-2 structure published recently by Umeda et al. (ref #25). However, as also pointed out by reviewer #1, we believe that the CD9 - EWI-F structure adds new important information to understand the molecular mechanism underlying the assembly of tetraspanin-enriched microdomains. Notably, the different conformations of the CD9 - EWI-F complex observed in the cryo-EM data provide structural biology evidence for the dynamic nature of the interaction between a tetraspanin and a partner protein, which is consistent with a wealth of prior biochemical data. Guided by the distinct shape of the $CD9_{EC2}$ - 4C8 densities, we were able to distinguish a range of straight to bent conformations of the complex. CD9 regions that represent known tetraspanin homo-dimerization sites, orient away from EWI-F and are available for interactions. Thus, combining our structural data with previous biochemical interaction data allowed for the generation of a long-awaited model for the assembly of tetraspanin-microdomains at the molecular level. We believe that these implications for TEM assembly will stimulate new, innovative research into the molecular principles that govern the function of tetraspanins.

[2.2] As such it may be acceptable for publication. In this case, the authors should improve the quality of Figs. 3D and 4D.

Reply 2.2: Figures 3D and 4D depict raw cryo-electron microscopy images (micrographs). The protein complexes imaged in this study only contain light atoms (H, N, C, O, S). Therefore, the collected micrographs only reveal low-contrast images of protein particles, and, for a typical cryo-EM experiment, it is required to average particles from thousands of micrographs to obtain a 3-dimensional reconstruction. We would like to keep the raw micrographs in figures 3 and 4, as it will aid cryo-EM scientists in judging the quality of the data.

Reviewer #3

The work is technically well performed and clearly presented including methodological details. I just have a few minor comments:

[3.1] Page 4 and Figure S1: it is hard to see how a reliable affinity for 4E8 can be obtained from the cell binding data in S1A, as there is no indication of saturation. It would be good to at acknowledge that this is at best a rough estimate. Fortunately the data for this nanobody in purified situation seems solid.

Reply 3.1: The obtained affinities are indeed an ±estimation based on a non-linear regression curve fitting on the measured data, performed in triplicate. The text has been updated and now reads as "4C8 and 4E8 bind to purified, full-length CD9 as well as to endogenous CD9 expressed on HeLa cells with apparent binding affinities in the nanomolar range (Fig. S1A, B, C)". Next to that, a table stating the calculated K_Ds has been included as Fig. S1C.

[3.2] Page 6: Does the absence of micellar density for the EWI-F complex indicate flexibility of the extracellular domain relative to the TM? Does this happen because the classification focuses on the highly elongated Ig region?

Reply 3.2: These are indeed plausible assumptions. We observed highly heterogeneous, elongated particles in the micrograph shown in Fig. 3D, indicating inter-domain flexibility. If the alignment software focusses on certain Ig-like domains, other regions of the protein complex will be averaged out. An additional complexity with these elongated particles was to select an appropriate box size

for particle picking and particle extraction, because the particles differ greatly in size based on their orientation (fully elongated side-views vs. much smaller top-views). When taken together, the complex of CD9 with full-length EWI-F was unsuitable for high-resolution structure determination; the subsequent strategy using EWI-$F_{\Delta Ig1\text{-}5}$ resulted in globular particles with less flexibility (Fig. 4D), which allowed for a more detailed structural characterization of the complex.

[3.3] Page 8: "Recently, a cryo-EM density map has been reported..." - please reference here.

Reply 3.3: We added the appropriate reference to the sentence: “Recently, a cryo-EM density map has been reported of CD9 in complex with an EWI-F homolog, EWI-2 (Umeda *et al*, 2020).”

[3.4] Relatively little is known about how tetraspanins help to organize partner receptors into defined membrane domains, evidence for which has emerged from super-resolution light microscopy. Based on their structural analysis of the CD9-EWI-F complex, including the heterogeneity apparent in the cryo-EM structure, they propose a feasible concatenation model for higher order oligomerization of these complexes in the membrane. Obviously the model will need to be tested rigorously by mutational analysis, particularly the EWI Ig6 interface, but as it stands the paper is a significant contribution to the field of tetraspanins.

Reply 3.4: From the 8.6 Å cryo-EM data, the amino-acid residues that form the EWI-F Ig6 dimer interface can indeed not be distinguished. However, our data on CD9 in complex with full-length EWI-F (Fig. 3E) and previous cross-linking data (André et al. In situ chemical cross-linking on living cells reveals CD9P-1 cis-oligomer at cell surface - PMID: 19703604) support that EWI-F forms dimeric assemblies. Regarding the concatenation model, we therefore think that it will be of great interest to establish the putative CD9 - CD9 interactions (identified through biochemical approaches), that would link CD9 - EWI-F tetramers into higher assemblies, in the context of native membranes. However, investigating these transient interactions would require various non-trivial experiments and was therefore not within the scope of the current study.

September 3, 2020

RE: Life Science Alliance Manuscript #LSA-2020-00883-TR

Prof. Piet Gros
Utrecht University
Dept. of Chemistry
Crystal and Structural Chemistry
Utrecht 3584 CH
Netherlands

Dear Dr. Gros,

Thank you for submitting your revised manuscript entitled "Implications for tetraspanin-enriched microdomain assembly based on structures of CD9 with EWI-F". We would be happy to publish your paper in Life Science Alliance pending final revisions necessary to meet our formatting guidelines.

In addition to the points listed below, please also address the following,
-please make sure that the author list in the manuscript and our system match
-please use the [10 author names, et al.] format in your references (i.e. limit the author names to the first 10)
-please double-check your callouts and figures: add a callout for Figure S4A, C, D in your main manuscript text; you have a callout for Figure 2 C,D but these are neither part of the figure legend nor the figure
-please move the Supplementary Note 1 to before the Tables section in the manuscript text

If you are planning a press release on your work, please inform us immediately to allow informing our production team and scheduling a release date.

To upload the final version of your manuscript, please log in to your account:
https://lsa.msubmit.net/cgi-bin/main.plex
You will be guided to complete the submission of your revised manuscript and to fill in all necessary information. Please get in touch in case you do not know or remember your login name.

To avoid unnecessary delays in the acceptance and publication of your paper, please read the following information carefully.

A. FINAL FILES:

These items are required for acceptance.

-- An editable version of the final text (.DOC or .DOCX) is needed for copyediting (no PDFs).

-- High-resolution figure, supplementary figure and video files uploaded as individual files: See our detailed guidelines for preparing your production-ready images, http://www.life-science-alliance.org/authors

-- Summary blurb (enter in submission system): A short text summarizing in a single sentence the study (max. 200 characters including spaces). This text is used in conjunction with the titles of papers, hence should be informative and complementary to the title. It should describe the context and significance of the findings for a general readership; it should be written in the present tense and refer to the work in the third person. Author names should not be mentioned.

B. MANUSCRIPT ORGANIZATION AND FORMATTING:

Full guidelines are available on our Instructions for Authors page, http://www.life-science-alliance.org/authors

We encourage our authors to provide original source data, particularly uncropped/-processed electrophoretic blots and spreadsheets for the main figures of the manuscript. If you would like to add source data, we would welcome one PDF/Excel-file per figure for this information. These files will be linked online as supplementary "Source Data" files.

Submission of a paper that does not conform to Life Science Alliance guidelines will delay the acceptance of your manuscript.

It is Life Science Alliance policy that if requested, original data images must be made available to the editors. Failure to provide original images upon request will result in unavoidable delays in publication. Please ensure that you have access to all original data images prior to final submission.

The license to publish form must be signed before your manuscript can be sent to production. A link to the electronic license to publish form will be sent to the corresponding author only. Please take a moment to check your funder requirements.

Reviews, decision letters, and point-by-point responses associated with peer-review at Life Science Alliance will be published online, alongside the manuscript. If you do want to opt out of having the reviewer reports and your point-by-point responses displayed, please let us know immediately.

Thank you for your attention to these final processing requirements. Please revise and format the manuscript and upload materials within 7 days.

Thank you for this interesting contribution, we look forward to publishing your paper in Life Science Alliance.

Sincerely,

Shachi Bhatt, Ph.D.
Executive Editor
Life Science Alliance

September 4, 2020

RE: Life Science Alliance Manuscript #LSA-2020-00883-TRR

Prof. Piet Gros
Utrecht University
Dept. of Chemistry
Crystal and Structural Chemistry
Utrecht 3584 CH
Netherlands

Dear Dr. Gros,

Thank you for submitting your Research Article entitled "Implications for tetraspanin-enriched microdomain assembly based on structures of CD9 with EWI-F". It is a pleasure to let you know that your manuscript is now accepted for publication in Life Science Alliance.

The final published version of your manuscript will be deposited by us to PubMed Central upon online publication.

Your manuscript will now progress through copyediting and proofing. It is journal policy that authors provide original data upon request.

Reviews, decision letters, and point-by-point responses associated with peer-review at Life Science Alliance will be published online, alongside the manuscript. If you do want to opt out of having the reviewer reports and your point-by-point responses displayed, please let us know immediately.

IMPORTANT: If you will be unreachable at any time, please provide us with the email address of an alternate author. Failure to respond to routine queries may lead to unavoidable delays in publication.

Scheduling details will be available from our production department. You will receive proofs shortly before the publication date. Only essential corrections can be made at the proof stage so if there are any minor final changes you wish to make to the manuscript, please let the journal office know now.

DISTRIBUTION OF MATERIALS:
Authors are required to distribute freely any materials used in experiments published in Life Science Alliance. Authors are encouraged to deposit materials used in their studies to the appropriate repositories for distribution to researchers.

You can contact the journal office with any questions, contact@life-science-alliance.org

Congratulations on a very nice paper. I hope you are pleased with how the manuscript was handled editorially. We look forward to future exciting submissions from your lab.

Sincerely,

Shachi Bhatt, Ph.D.
Executive Editor
Life Science Alliance
